# Microbial community and metabolic pathway succession driven by changed nutrient inputs in tailings: effects of different nutrients on tailing remediation

**DOI:** 10.1038/s41598-017-00580-3

**Published:** 2017-03-28

**Authors:** Mingjiang Zhang, Xingyu Liu, Yibin Li, Guangyuan Wang, Zining Wang, Jiankang Wen

**Affiliations:** National Engineering Laboratory of Biohydrometallurgy, General Research Institute for Nonferrous Metals, No. 2 Xinjiekouwai Street, Beijing, 100088 China

## Abstract

To solve the competition problem of acidophilic bacteria and sulfate-reducing bacteria in the practical application of mine tailing bioremediation, research into the mechanisms of using different nutrients to adjust the microbial community was conducted. Competition experiments involving acidophilic bacteria and sulfate-reducing bacteria were performed by supplementing the media with yeast extract, tryptone, lactate, and glucose. The physiochemical properties were determined, and the microbial community structure and biomass were investigated using MiSeq sequencing and qRT-PCR, respectively. Four nutrients had different remediation mechanisms and yielded different remediation effects. Yeast extract and tryptone (more than 1.6 g/L) promoted sulfate-reducing bacteria and inhibited acidophilic bacteria. Lactate inhibited both sulfate-reducing and acidophilic bacteria. Glucose promoted acidophilic bacteria more than sulfate-reducing bacteria. Yeast extract was the best choice for adjusting the microbial community and bioremediation, followed by tryptone. Lactate kept the physiochemical properties stable or made slight improvements; however, glucose was not suitable for mine tailing remediation. Different nutrients had significant effects on the abundance of the second enzyme of the sulfate-reducing pathway (*p* < 0.05), which is the rate-limiting step of sulfate-reducing pathways. Nutrients changed the remediation effects effectively by adjusting the microbial community and the abundance of the sulfate-reducing rate-limiting enzyme.

## Introduction

According to the official statistics from the state administration of work safety in 2012, approximately 12,273 mine tailings have generated eight billion tons of mine waste in China^1^. Mine tailings usually contain high heavy metal concentrations^2^. With the presence of both oxygen and water, mine tailings usually oxidize naturally and produce sulfate, metal iron, and proton acidity^3^. The natural oxidation process can decrease the pH and promote the growth of autotrophic iron- and sulfur-oxidizing acidophilic bacteria. At the same time, acidophilic bacteria can accelerate the release of metal-rich acid mine drainage (AMD)^4^ and promote the formation of a vicious pollution cycle in the acidic mine tailings^5^. A method to address mine tailing pollution is urgently needed worldwide.

Recently, a large amount of research has focused on the microbial remediation of heavy metal pollution in abandoned mine tailings, acid mine drainage, and groundwater^6–8^. In particular, remediation by sulfate-reducing bacteria (SRB) is attracting more attention^9^. In the SRB remediation process, sulfate is reduced to hydrogen sulfide, the dissolved metal ions are then precipitated as metal sulfides, and the concentration of heavy metal ions in solution decreases^10, 11^. At the same time, hydrogen ions are consumed, and the pH increases. The changes of the tailing parameters caused by SRB are beneficial for not only the formation of a virtuous cycle but also the restoration of vegetation^12^. Therefore, the use of SRB for tailing remediation could become an attractive biotechnology in the future.

Because of the inherent existence of autotrophic iron- and sulfur-oxidizing acidophilic bacteria in mine tailings, the bioremediation process with SRB is also the competition process between SRB and acidophilic bacteria. Environmental deterioration emerges when acidophilic bacteria become dominant in the tailings; by contrast, the mine tailing environment can improve when SRB become dominant. Reasonable adjustments of the microbial community structure are very important for the bioremediation of tailings. The current research in SRB remediation mainly focuses on researching the remediation capabilities of SRB^6, 13^ and neglects the inherent existence and competition of acidophilic bacteria in mine tailings. Studies assessing microbial competition and microbial community adjustment are both meaningful and needed for the practical remediation application of SRB.

Most acidophilic bacteria are autotrophs, which are capable of manufacturing complex organic compounds from simple inorganic sources, such as carbon dioxide, water, and nitrates^14^. However, acidophilic bacteria cannot utilize organic substances; some acidophilic bacteria can even be inhibited or killed by some organic substances. By contrast, the growth of heterotrophic SRB depends entirely on complex organic substances and can be promoted by organic substances. According to the differences in nutritional utilization characteristics between autotrophic bacteria and heterotrophic SRB, the microbial community structure might be adjusted to have higher bioremediation efficiency. This study seeks to investigate the effects of different nutrients on the microbial community, sulfur metabolism and mine tailing remediation; to analyze the correlations between nutrients, the microbial community and mine tailing remediation; and to determine the mechanism of action of different nutrients on mine tailing remediation.

## Results and Discussion

### Physiochemical properties of different experimental groups

**Figure 1:**
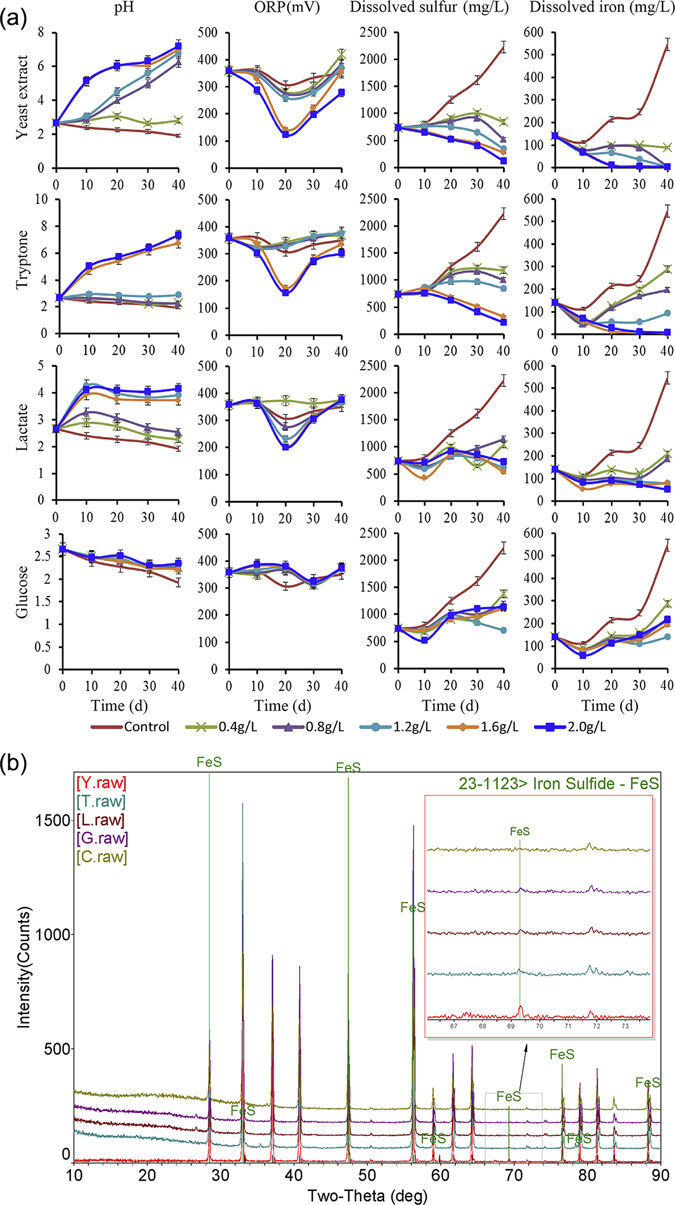
Remediation effects in the different groups. The physiochemical properties of different bioremediation systems **(a)**. Rows represent different nutrients, columns show different physicochemical parameters: pH, ORP, dissolved sulfur and dissolved iron. Identification of reaction products by XRD **(b)**. Yeast extract-supplemented group (Y), Tryptone-supplemented group (T), Lactate-supplemented group (L), Glucose-supplemented group (G), control group (C).

Solution physiochemical properties of different experimental groups are summarized in Fig. 1a. Solution pH results revealed that yeast extract at 0.4 g/L inhibited the decrease in pH, whereas yeast extract at more than 0.8 g/L increased the solution pH to neutral. Tryptone also elevated the solution pH to neutral when tryptone was present at more than 1.6 g/L, but tryptone only inhibited pH reduction rather than raising the pH when its concentration was less than 1.2 g/L. Lactate increased the solution pH, but the pH could only be raised to 4–5. Glucose inhibited pH reduction rather than raising the pH. The solution pH strongly impacted the dynamics of dissolved heavy metals^15^, and the stability of metals correlated with the increasing pH^16^. Therefore, yeast extract was the best for metal stability, followed by tryptone, lactate, and glucose.

Oxidization-reduction potential (ORP) is important not only for the formation and the mobilization of minerals^17^ but also for microbial communities^18^. In the control group, the ORP decreased slightly over the period from 10–30 days. In glucose-supplemented flasks, the ORP also experienced a smaller fluctuation over the period from 20–30 days, but the fluctuation had no correlation with the glucose concentration. This result implied that the decrease in ORP might not be caused by glucose. The decreases in the ORP in the control and glucose-supplemented groups resulted from anaerobic conditions because some acidophilic bacteria and SRB can reduce sulfate and decrease the ORP with anaerobic conditions^19^. In yeast extract-, tryptone-, and lactate-supplemented flasks, the ORP drastically decreased in the 10–20 day period, indicating that yeast extract, tryptone, and lactate can decrease the ORP. However, the ORP rebounded and stabilized after 20 days, suggesting that these results may be related to the microbial growth cycle. The ORP changes observed in closed reactors mainly resulted from the oxidation-reduction reaction of acidophilic bacteria and SRB. Therefore, the ORP could be taken as an indicator indirectly reflecting the ratio, the activity changes, and the competition situation of acidophilic bacteria and SRB^20^.

The dissolved sulfur in solution mainly included oxidized sulfur (SO_4_^2−^, SO_3_^2−^) and reduced sulfur (S^2−^). In the sulfate-reducing process, sulfate can be reduced to sulfion, and sulfion can then be precipitated with metal^10, 21, 22^. Because of the lower solubility of metal sulfides, S^2−^ in solution usually is ignored. As a result, the dissolved sulfur could roughly be considered oxidized sulfur. Dissolved sulfur and dissolved iron usually correlate with mine tailing oxidizing and pollution; therefore, dissolved sulfur and dissolved iron could be taken as indicators of mine tailing pollution. Yeast extract and tryptone could significantly decrease the dissolution of sulfur and iron (*p* < 0.05). These results indicated that the supplemented yeast extract and tryptone could remove the dissolved sulfur and iron and could improve the tailing environment significantly. Lactate and glucose inhibited the dissolution of sulfur and iron, but there were no significant decreases in the dissolved sulfur and iron. These results indicated lactate and glucose could only inhibit deterioration rather than immobilize sulfur and iron.

Ferrous sulfide is the product of the sulfate-reducing process; therefore, the output of ferrous sulfide can be taken as an important indicator to test any bioremediation effect. In this experiment, the X-ray diffraction (XRD) results (Fig. 1b) indicated that ferrous sulfide was generated in all four organic matter-supplemented groups, while no ferrous sulfide was generated in the control group. Ferrous sulfide generation results in decreased dissolution of sulfur and iron^10, 22^, and all four of these types of organic matter promote bioremediation. Among these four groups, the yeast extract-supplemented group had the maximum yield of ferrous sulfide.

### Effects of different nutrients on the microbial growth cycle, biomass and alpha diversity

Nutrients also impacted the microbial growth phase and total biomass (Fig. S1). The effects of yeast extract, tryptone, and glucose were similar compared with those of the control. Supplementation of yeast extract, tryptone, and glucose significantly elevated the microbial biomass (*p* < 0.05); furthermore, the biomass correlated with the nutrient concentration (*p* < 0.05). However, excessively high nutrient concentrations led microbial communities to prematurely enter the decline phase and shortened the stable phase^23^. These results indicated that an excessively high nutrient concentration was unfavorable for microbial stability. Compared with that of the control, lactate also contributed to increasing microbial biomass, but the contribution of lactate to the microbial biomass was less than the other three nutrients.

The alpha diversity results are shown in Fig. S2. Yeast extract and tryptone increased the Chao1 index (microbial richness) and the Shannon index (microbial richness and uniformity) compared with those of the control group. Because of the presence of heavy metals and lower pH values in mine tailings, most microbes can hardly survive in this poor environment. Acidophilic bacteria were the dominant microbes^24^, and the microbial richness and uniformity were comparatively low. The increase of the Chao1 index and the Shannon index in the yeast extract- and tryptone-supplemented groups implied improvements in the mine tailing environment. Lactate elevated the Shannon index and reduced the Chao1 index, which implied that lactate restrained the original dominant bacteria. The decrease of the Shannon index in the glucose-supplemented group implied that the microbial community involved was more unbalanced.

### Microbial community succession is driven by different nutrient inputs

**Figure 2:**
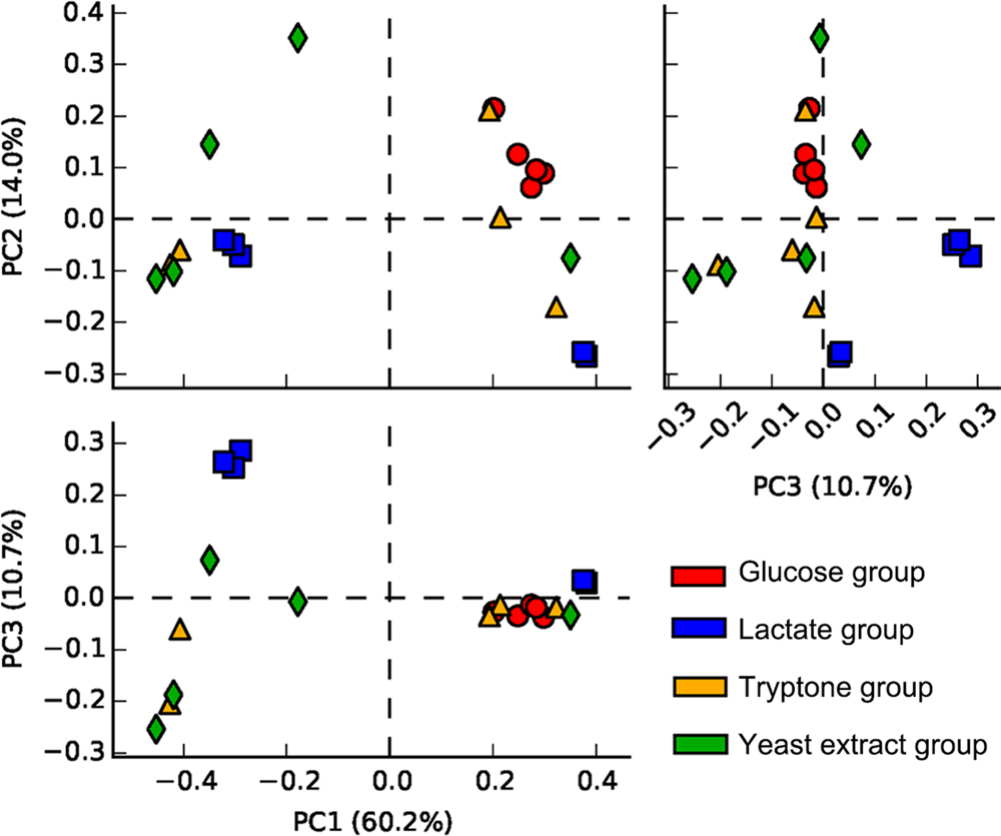
Principal component analysis results for the microbial community.

Different nutrients can strongly affect and change microbial community compositions^22, 25^. Principal component analysis (PCA) results for the microbes (Fig. 2) revealed that samples from the yeast extract-supplemented group had the maximum distance, followed by the tryptone and lactate groups. These results indicated that the yeast extract had the largest effects on the microbial community, followed by tryptone and lactate; glucose had a minimal impact on the principal microbes. Therefore, yeast extract is the most effective nutrient to change this microbial community.

**Figure 3:**
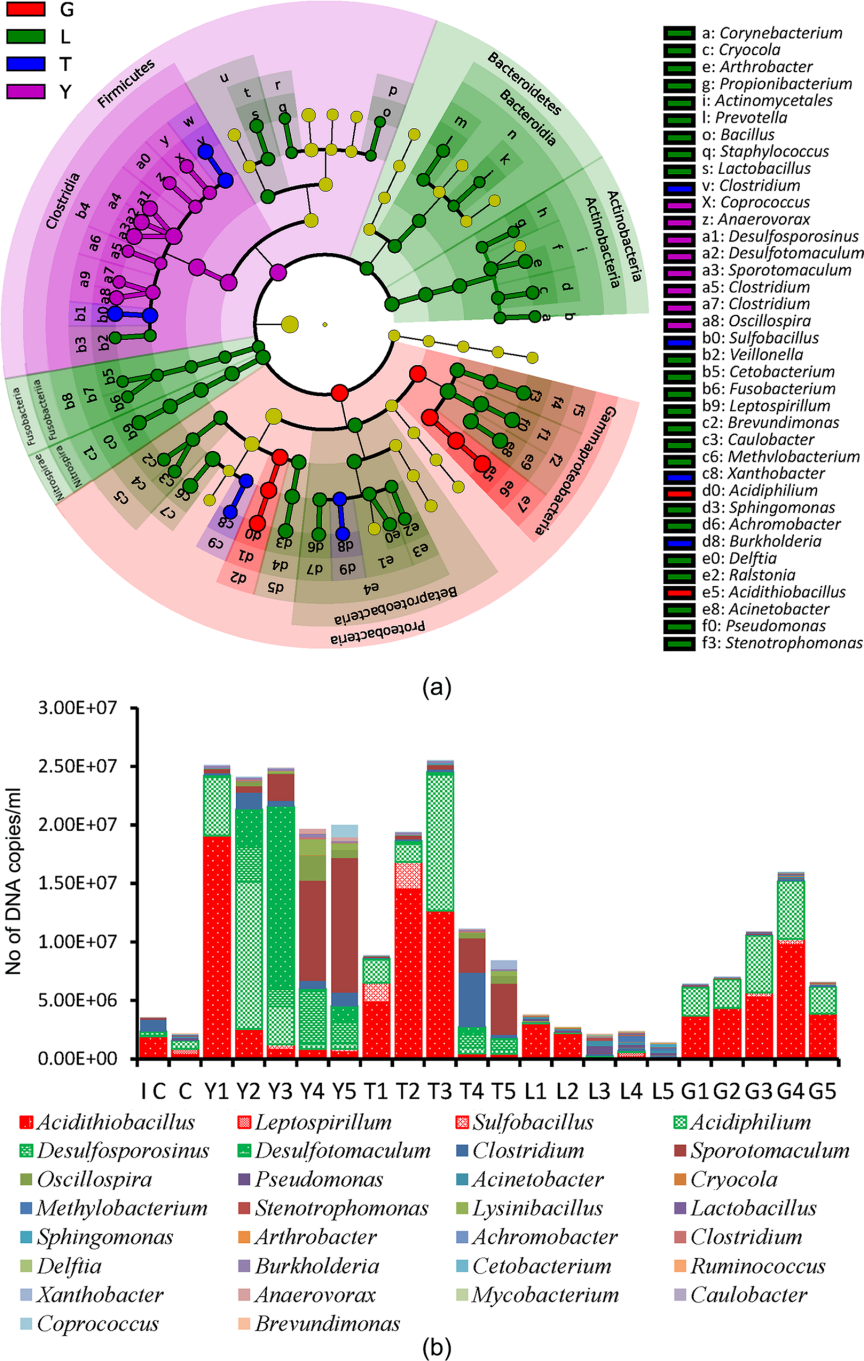
Effects of different nutrients on the microbial community. Linear discriminant analysis effect size (LEfSe) results of the main microbes (**a**). The microbial community and the biomass of the main microbes after 40 days of remediation. IC is the initial control sample.

A linear discriminant analysis coupled with effect size analysis (LEfSe) was used to determine organisms most likely to explain differences between experimental groups. The LEfSe results (Fig. 3a) revealed yeast extract can promote the growth of the Firmicutes phylum, including *Desulfosporosinus* and *Desulfotomaculum*. Glucose promotes the growth of the Proteobacteria phylum, especially for the key acidophilic bacteria, *Acidithiobacillus*, present in mine tailings. In this sense, glucose is adverse for the immobilization of heavy metals. However, glucose also promotes the growth of *Acidiphilium*, which can reduce Fe(III) to Fe(II)^26^, which is beneficial for the remediation of mine tailings.

The microbial community structure after bioremediation for forty days is summarized in Fig. 3b. With increasing yeast extract concentration, the percentage of autotrophic acidophilic bacteria decreased, and the heterotrophic bacteria percentage increased significantly (*p* < 0.05). When the yeast extract concentration exceeded 0.8 g/L, the percentage of heterotrophic bacteria began to exceed that of autotrophic acidophilic bacteria. When the concentration of yeast extract exceeded 1.2 g/L, SRB began to replace acidophilic bacteria and became the dominant bacteria. However, with increasing yeast extract concentration, the growth of uncorrelated bacteria was also promoted, which was unfavorable. These results indicated that yeast extract could effectively adjust the microbial community but that too much yeast extract was not necessary.

Lower concentrations (less than 1.2 g/L) of tryptone promoted growth of not only SRB and the uncorrelated heterotrophic bacteria but also acidophilic bacteria. These results indicated that tryptone at lower concentrations could not effectively adjust the microbial community. When the concentration of tryptone exceeded 1.6 g/L, tryptone began to inhibit the growth of acidophilic bacteria and to significantly promote the growth of SRB and other heterotrophic bacteria (*p* < 0.05). These results indicated that only higher concentrations (more than 1.6 g/L) of tryptone could effectively adjust the microbial community.

With increasing lactate concentration, acidophilic bacterial growth was inhibited significantly (*p* < 0.05), but lactate’s promotion of SRB was not significant (*p* > 0.05). The mechanism lactate employs to adjust the microbial community mainly depends on suppression of acidophilic bacteria, different from yeast extract and tryptone.

Glucose exhibited more promotion of acidophilic bacteria than SRB; therefore, glucose was unfavorable for structural adjustment of the microbial community. Most acidophilic bacteria are autotrophic bacteria. Autotrophic bacteria usually grow with carbon dioxide as the carbon source^14^, rarely utilize organic substances and are sensitive to organics^27^. However, there were more acidophilic bacteria in the glucose-supplemented sample than in the control, confirming that glucose promoted the growth of acidophilic bacteria. This conclusion was consistent with reports on the capacity of acidophilic bacteria to utilize glucose^28–30^. There were more acidophilic bacteria, but less sulfur and iron dissolved in the glucose-supplemented sample than in the control. These observations may relate to improved sulfate removal efficiency in the glucose-supplemented group because the utilization of glucose by SRB can not only promote the growth of SRB but can also improve the sulfate removal efficiency^23^.

### Correlations of nutrients, microbes and physiochemical properties

The heat map of the top 30 genera is shown in Fig. S3. The heat map results easily assigned 20 experimental treatments to two big groups, and this classification according to the microbial community was consistent with the remediation effects, the left- and right-side groups had poor and good remediation effects, respectively. This result implies that the remediation effects and the microbial community had some correlation.

**Figure 4:**
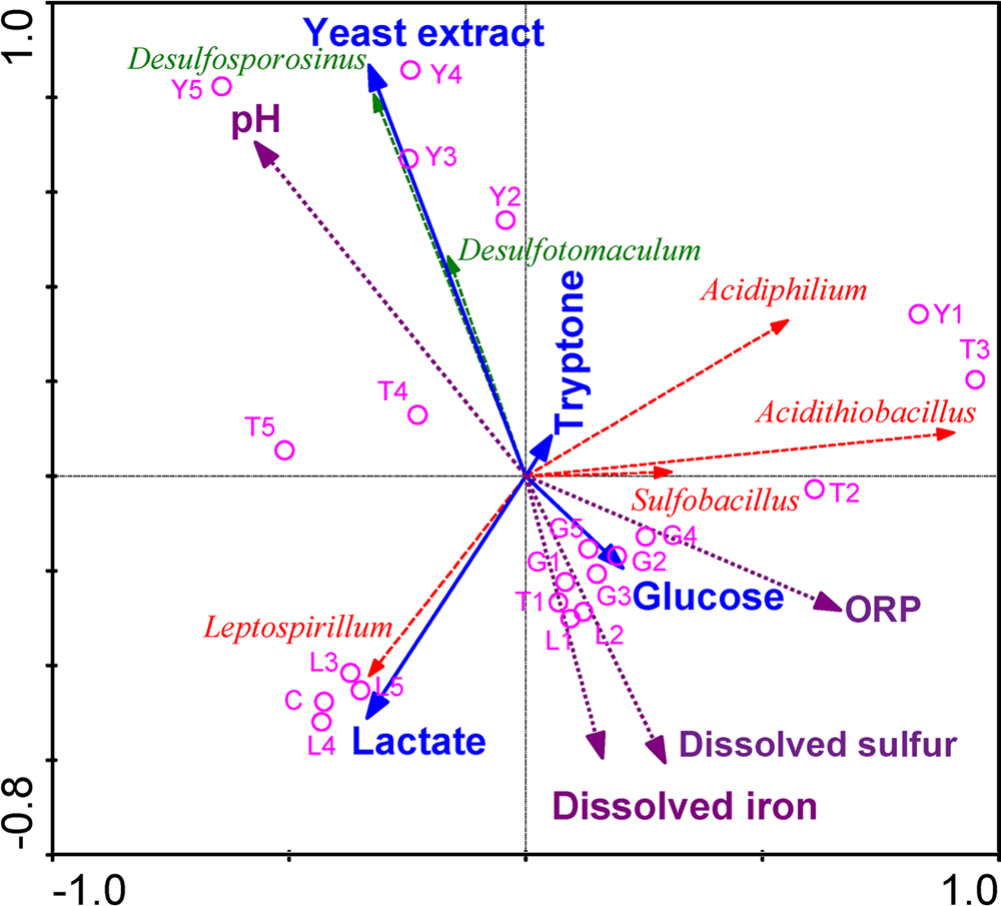
Redundancy analysis. Redundancy analysis triplots show the relationships between the different nutrients, the microbial community and the physiochemical properties. Nutrients are shown with solid lines and solid arrows; experiments with different nutrients and concentrations are shown with empty circles; microbes are shown with dashed lines; physiochemical properties are shown with dotted lines and solid arrows.

The correlations of nutrients, microbes, and physiochemical properties were determined using the redundancy analysis (RDA) method (Fig. 4). RDA analysis indicated that the first two axes explained 96.0% of the species–environment correlations. Yeast extract showed a notably positive correlation with SRB and solution pH (*p* < 0.05) but showed a notably negative correlation with dissolved sulfur and dissolved iron (*p* < 0.05). Additionally, there appeared to be negative correlations with *Leptospirillum*, *Sulfobacillus*, *Acidithiobacillus*, and the ORP. These correlations implied that yeast extract mainly promoted SRB growth rather than inhibited acidophilic bacteria to adjust the microbial community and remediate the tailing. Tryptone also exhibited positive correlations with SRB and *Acidiphilium* but exhibited a negative correlation with *Leptospirillum*; these results implied that tryptone could also promote the growth of SRB and *Acidiphilium* while inhibiting growth of *Leptospirillum*. Furthermore, tryptone at the lower concentration also promoted the growth of *Acidithiobacillus* and *Sulfobacillus*, though the remediation effect was inferior to that of yeast extract. Lactate showed more negative correlations with *Acidiphilium*, *Acidithiobacillus*, and *Sulfobacillus* and a less negative correlation with SRB. Because the inhibition of lactate on oxidizing acidophilic bacteria was stronger than on the SRB, lactate exhibited more inhibition on the deterioration than on the bioremediation. Lactate also exhibited some remediation effects compared with those of the control. Glucose showed a positive correlation with oxidizing acidophilic bacteria and a negative correlation with SRB. These results implied that adjusting the microbial community with glucose was not helpful for the remediation of tailing.

### Metabolism pathway succession is driven by different nutrient inputs

**Figure 5:**
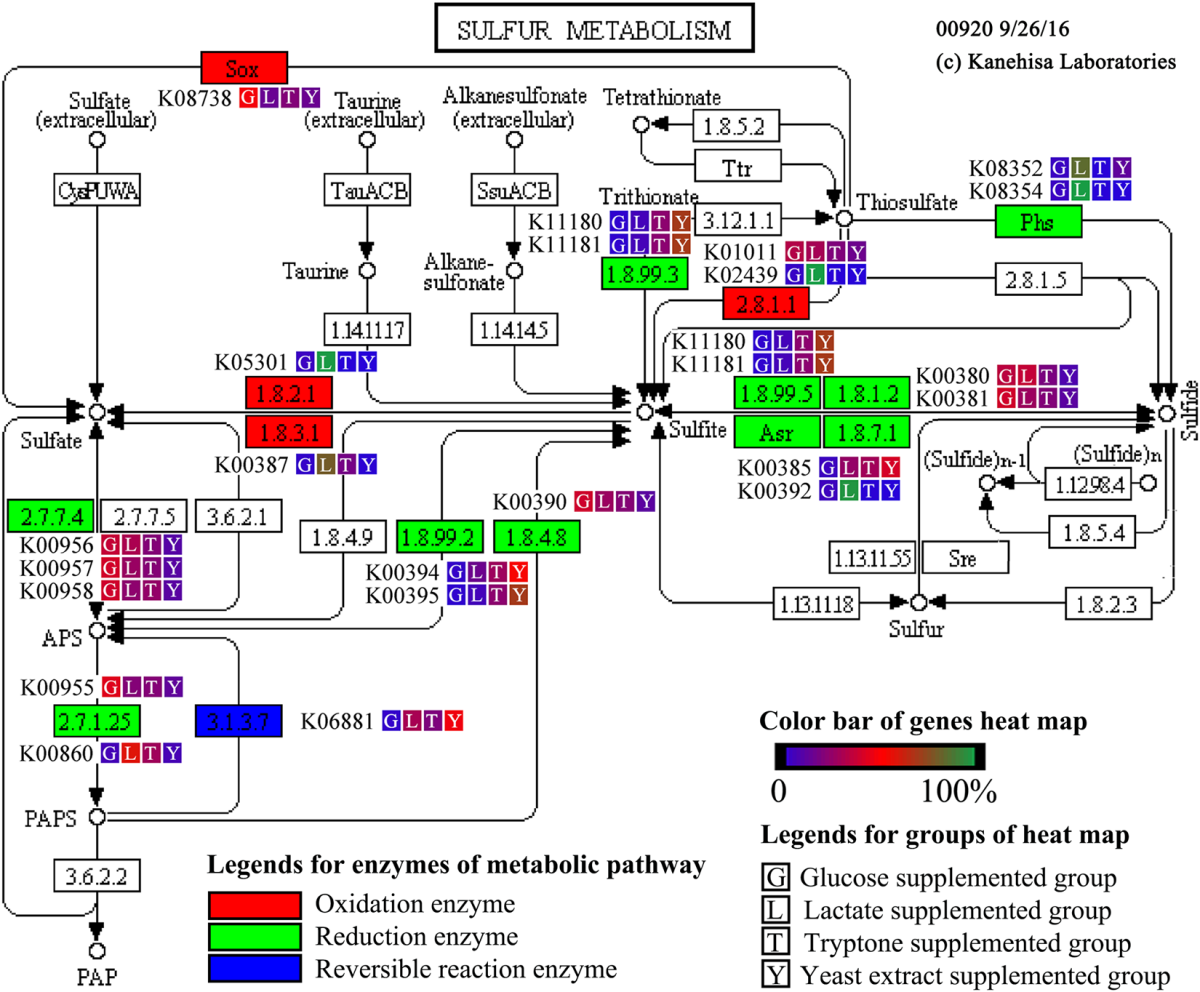
Differences in the sulfur metabolic pathway in the different groups, sulfur metabolic pathway is obtained by KEGG.

Figure 5 and Fig. S4, respectively, illustrate the differences in the sulfur metabolism pathway and the significantly different genes (*p* < 0.05) in the different groups. The expression products of k00955, k00956, k00957, and k00958 are isozymes and encode 3′-phosphoadenosine 5′-phosphosulfate synthase. 3′-phosphoadenosine 5′-phosphosulfate synthase catalyzes the reaction involving sulfate to produce adenosine 5′-phosphosulfate (APS) or 3′-phosphoadenosine 5′-phosphosulfate (PAPS), which is the first reaction in sulfate reduction^31, 32^. These four genes had no significant differences in the different groups (*p* > 0.05). In addition, these results indicated that there were no significant effects on the metabolism of sulfate to APS or PAPS among the different groups.

k00394 and k00395 encode adenylylsulfate reductase, which catalyzes APS to sulfite^33, 34^, the second reaction of sulfate reduction^31^. There was a higher abundance of k00394 and k00395 in the yeast extract and tryptone groups than in the glucose and lactate groups. Particularly in the glucose group, very little k00394 and k00395 were detected. These results indicated that the adenylylsulfate reductase enzymes encoded by k00394 and k00395 might impact sulfate reduction; furthermore, this step might be one of the rate-limiting steps of sulfate reduction.

The genes k11180, k11181, k00380, k00381, k00385, k01001, and k00392 encode sulfite reductase^35, 36^, which can catalyze sulfite to sulfide, the third reaction of sulfate reduction^31^. The differences between k11180, k11181, k00380, and k00392 were significant (*p* < 0.05); k11180 and k11181 had more copy numbers in the yeast extract and tryptone groups, and k00380 and k00392 had more copy numbers in the lactate group and glucose groups. However, the differences between k00381, k00385, and k01001 were not significant (*p* > 0.05) in the different groups. As a result, there are no significant effects on the metabolism from sulfite to sulfide among the different groups.

Different nutrients had no significant effects on the first and the third enzymes involved in the process of sulfate reduction. However, the second enzyme differed significantly in the different groups; therefore, the second enzyme is the rate-limiting step of sulfate reduction under these conditions. Some nutrients, such as yeast extract, can promote sulfate reduction by adjusting the microbial community and increasing the available amount of the second enzyme.

### Remediation experiment of tailings on the natural conditions

The microbial community of key microbes (Fig. S5) showed acidophilic bacteria were the predominant bacteria of tailings, and SRB only accounted for a small percentage on the natural conditions. *Thiobacillus* percentage increased in the control group, however, *Desulfotomaculum* percentage increased in yeast extract sprayed group. Physical and chemical properties of exudate (Table 1) revealed pH elevated, ORP and the concentration of S, Fe, Cu, Zn, and Pb decreased in yeast extract sprayed group. These results implied yeast extract can promote the growth of *Desulfotomaculum* and adjust the microbial community of tailings effectively, can improve the physical and chemical properties of exudate on the natural conditions.

**Table 1:** Physical and chemical properties of exudate between control group and yeast extract sprayed group.

Physical and chemical properties	Control group	Yeast supplemented group
pH	3.67 ± 0.06	7.50 ± 0.08
ORP (mV)	574 ± 8.5	386 ± 11.3
S (g/L)	4.09 ± 0.02	2.84 ± 0.01
Fe (mg/L)	256.3 ± 0.6	108.7 ± 0.9
Cu (mg/L)	20.77 ± 0.02	0.107 ± 0.01
Zn (mg/L)	1.657 ± 0.03	0.017 ± 0
Pb (mg/L)	0.137 ± 0	0.027 ± 0

## Conclusions

Four nutrients had different remediation mechanisms and demonstrated different remediation effects. Yeast extract and high concentrations of tryptone (more than 1.6 g/L) could promote sulfate-reducing bacterial growth and inhibit acidophilic bacterial growth. Lactate could simultaneously inhibit both sulfate-reducing and acidophilic bacteria. Glucose could promote acidophilic bacteria more than sulfate-reducing bacteria. Yeast extract was the best choice for adjusting the microbial community and bioremediation, followed by tryptone, because these two nutrients could improve the mine tailing environment by effectively adjusting the microbial community. Lactate could keep the physiochemical properties stable or make small improvements, whereas glucose was not suitable for mine tailing remediation. Different nutrients had no significant effects on the first and the third enzymes involved in sulfate reduction; however, the second enzyme differed significantly among the different groups, indicating that the second enzyme is the rate-limiting step of sulfate reduction under these conditions. Nutrients can alter the abundance levels of sulfate-reducing rate-limiting enzymes by adjusting the microbial community and, consequently, can change the remediation effects. On the natural conditions, yeast extract also can promote the growth of *Desulfotomaculum* of tailings, and improve the physical and chemical properties of exudate.

## Materials and Methods

### Laboratory-scale experiments

Experiments were performed in sealed 250-mL flasks with 150 mL of modified 0 K medium (0.1 g/L KCl, 0.5 g/L K_2_HPO_4_, 0.5 g/L MgSO_4_·7H_2_O, 0.01 g/L Ca(NO_3_)_2_, pH 2.8). Yeast extract (Y), tryptone (T), lactate (L), and glucose (G) were supplemented in different experimental groups. The concentrations of the nutrients were 0.4 g/L, 0.8 g/L, 1.2 g/L, 1.6 g/L, and 2.0 g/L for solutions numbered 1, 2, 3, 4, and 5, respectively; the control was 0 g/L. Each treatment was replicated twice. For each treatment, about 5.0 × 10^8^ oxidizing acidophilic bacteria and 2% (v/v) pyrite were added to simulate the mine tailing environment, and about 5.0 × 10^8^ SRB were added for remediation. Microbes were statically cultivated at 30 °C for 40 days, and samples were collected and analyzed every 10 days for 40 days.

### Remediation experiment of tailings on the natural conditions

Remediation experiments on the natural conditions were performed in GuangXi XinXing tailings (Fig. S6). 2,000 m^2^ tailings were taken as remediation group, spraying 1,000 t yeast extract solution (1.2 g/L) in 10 days. Another 2,000 m^2^ tailings were taken as control group, spraying the same amount of water in 10 days. At the beginning of this experiment and after spraying 40 days, five tailings samples from each group were collected to determine the microbial community respectively, and five exudate samples from each group were collected to determine the physical and chemical properties respectively.

### Physicochemical analysis of the samples

The pH and ORP were determined using a pH and ORP electrode^37^. The dissolved iron and sulfur were analyzed using inductively coupled plasma atomic emission spectroscopy (ICP-OES)^38, 39^ after the samples were filtered with super membrane filters (0.2 µm pore size, Sigma-Aldrich, MO, USA). The product was determined by XRD and analyzed by MDI Jade 6^40^.

### DNA extraction and real-time PCR

Whole DNA samples were extracted using the E.Z.N.A. bacterial DNA kit (OMEGA, D3350-01) according to the manufacturer’s instructions. The real-time PCR assay of the total microbial biomass used the universal primers (F: GTAGTCCMSGCYSTAAACGATG, R: AGCTGRCGACRRCCATGCA), which match 16S rDNA sequences from bacteria and archaea^41^. Real-time PCR was performed with a Rotor-Gene 6000 (Corbett Research) using SYBR Green I (Applied Biosystems) according to the manufacturer’s instructions, all tests were conducted in triplicate.

### MiSeq sequencing and data processing

The 16S rRNA genes were sequenced with the 340F/805R primer set (340F: CCTACGGGNGGCWGCAG, 805R: GACTACHVGGGTATCTAATCC), which amplifies the V3-V4 region of the 16S rDNA gene^42^. Sequencing was conducted on an Illumina MiSeq high-throughput sequencing technology platform^43, 44^ by Sino Geno Max (Beijing, China). All of the 16S raw data were deposited in the NCBI Sequence Read Archive (SRA) under the submission ID SUB1935448 and BioProject ID PRJNA342734.

Paired-end reads of the original DNA fragments from high-throughput sequencing were merged using FLASH^45^ and were assigned to each sample according to the unique barcodes. The 16S rRNA genes were processed using the open-source software QIIME^46, 47^, and in-house Perl scripts were used to analyze alpha (within samples) and beta (among samples) diversity. The Chimera Slayer tool was used for chimera detection^48^, and the CD-HIT package^49^ and the QIIME script “pick_de_novo_otus.py”^46^ were then used to pick operational taxonomic units (OTUs) to construct an OTU table. Sequences with ≥ 97% similarity were assigned to the same OTUs^50^. Representative sequences for each OTU were picked, and the Ribosomal Database Project (RDP) classifier was used to annotate taxonomic information for each representative sequence^51^. PCA and heat map and box plot construction were performed using STAMP software^52^. LEfSe was used to explore potential biomarkers for distinguishing taxonomy^53^. A linear discriminant analysis (LDA) threshold score (0.05) for the factorial Kruskal-Wallis test and the all-against-all multi-class analysis strategy were used to detect significantly different microbes^54^. Contribution rates of functional genes were predicted from PICRUSt^55^ through inputting the 16S rRNA OTU table. A sulfur metabolic pathway was constructed from input data to the Kyoto Encyclopedia of Genes and Genomes (KEGG) (http://www.genome.jp/kegg-bin/show_pathway?map=map00920&show_description=show)^56^.

### Statistical analysis

The physicochemical indexes were statistically analyzed with separate one-way analyses of variance (ANOVA). The correlations were analyzed with bivariate correlations. These statistical analyses were performed using SPSS 19.0 for Windows^57^. RDA was implemented with the CANOCO 4.5 software package^58^.

## Electronic supplementary material

Supplementary Figures.
