# Supplementary material for: Microbial community and metabolic pathway succession driven by changed nutrient inputs in tailings: effects of different nutrients on tailing remediation
Source: Sci Rep. 2017 Mar 28;7:474. doi: 10.1038/s41598-017-00580-3 (PMC5428726; doi:10.1038/s41598-017-00580-3)
Supplement: Supplementary file 1 — Supplementary Figures. [file 41598_2017_580_MOESM1_ESM.pdf]

Microbial community and metabolic pathway succession driven by changed nutrient inputs in tailings: effects of different nutrients on tailing remediation

Mingjiang Zhang, Xingyu Liu*, Yibin Li, Guangyuan Wang, Zining Wang, Jiankang Wen

National Engineering Laboratory of Biohydrometallurgy, General Research Institute for Nonferrous Metals, No. 2 Xinjiekouwai Street, Beijing, China 100088.

Corresponding author: *wellwoodliu@163.com, 8610-82241312

Fig caption:

Fig. S1 The qRT-PCR results of microbial biomass with universal primers.

Fig. S2 Alpha diversity index of microbial community

Fig. S3 The heat map of the key microbes in different experiment treatment

Fig. S4 Visualization the differences of sulfur metabolism genes that differed significantly (*p*<0.05) by box plot

Fig. S5 Microbial community of remediation experiment on the natural conditions

Fig. S6 Remediation experiment of tailings on the natural conditions

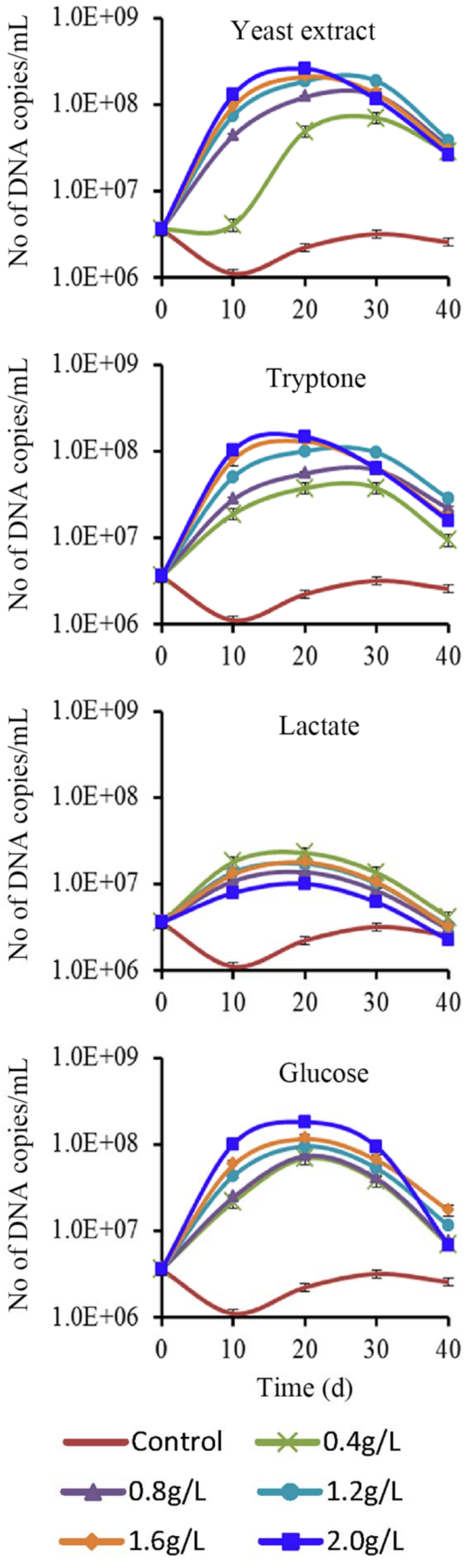

Fig. S1 The qRT-PCR results of microbial biomass with universal primers.

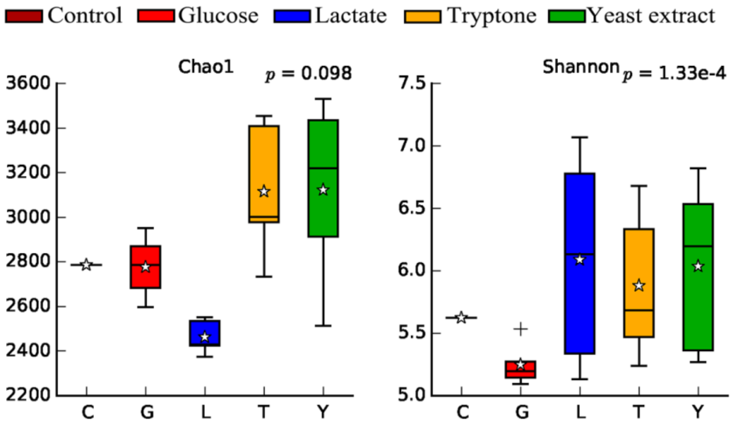

Fig. S2 Alpha diversity index of microbial community

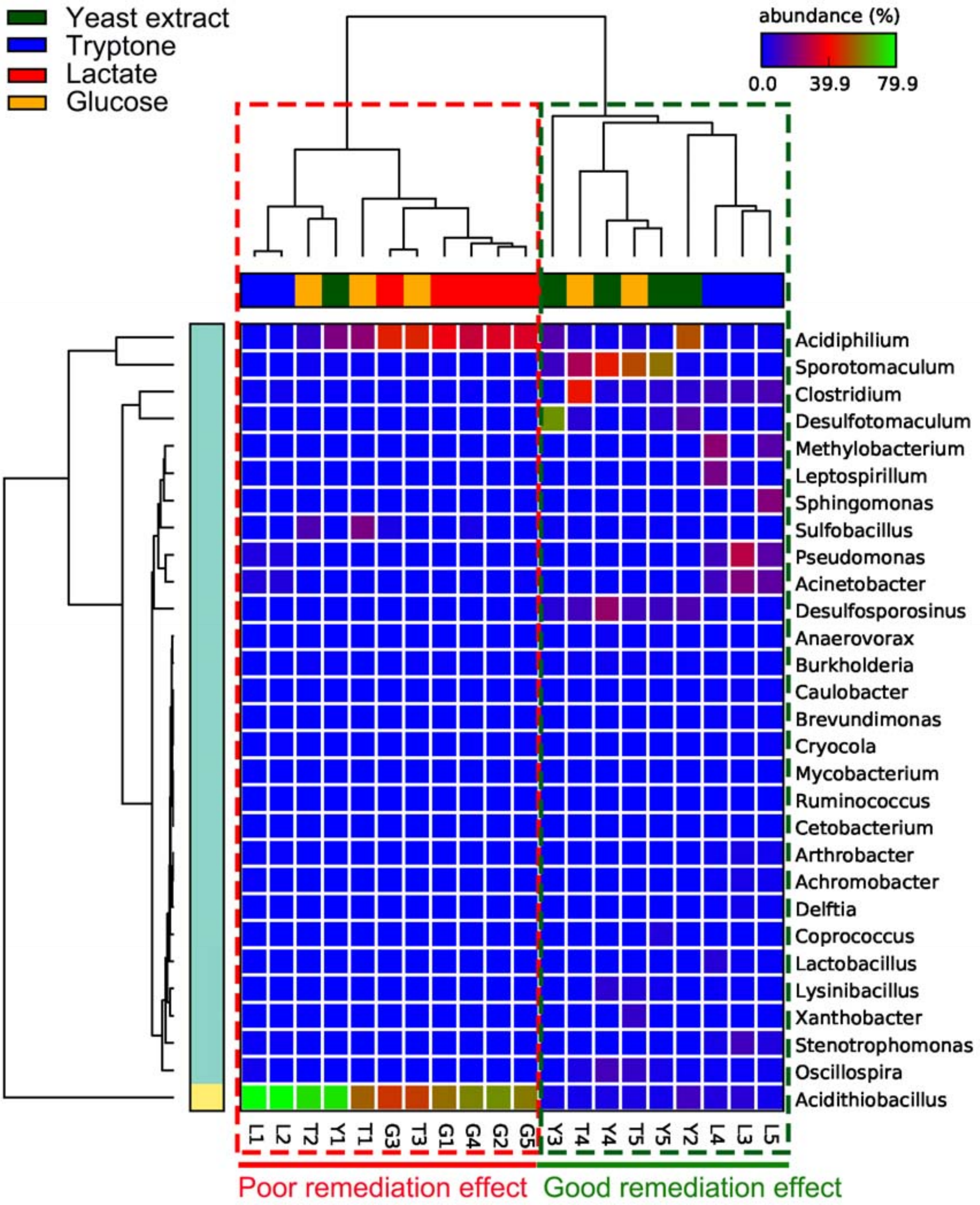

Fig. S3 The heat map of the key microbes in different experiment treatment

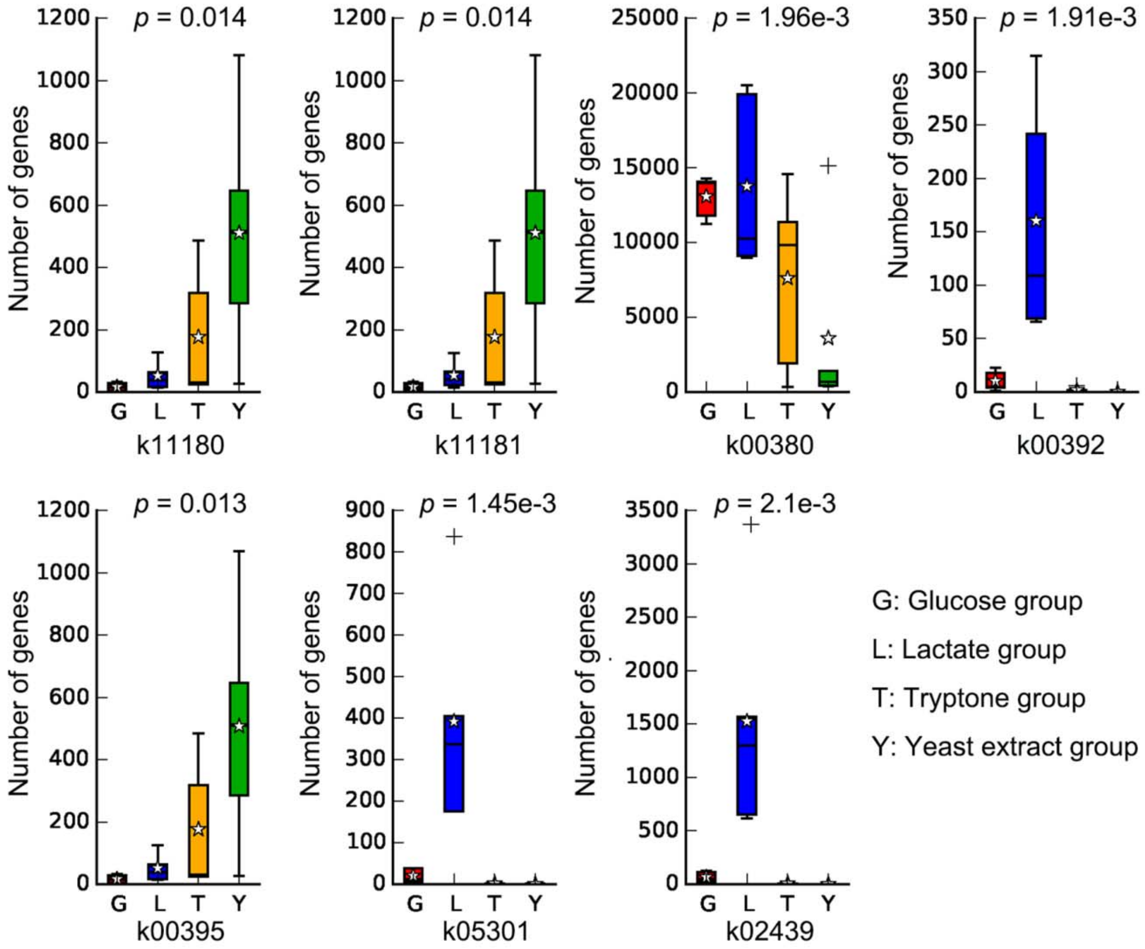

Fig. S4 Visualization the differences of sulfur metabolism genes that differed significantly (p<0.05) by box plot

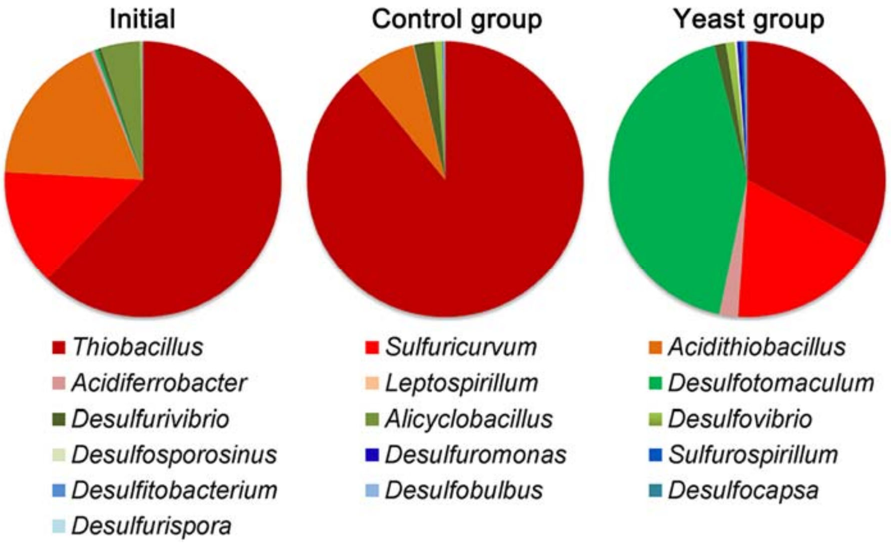

Fig. S5 Microbial community of remediation experiment on the natural conditions

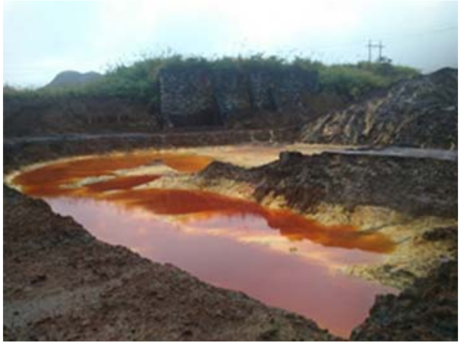

Fig. S6 Remediation experiment of tailings on the natural conditions
